# Supplementary figures and images for: Structural insights into Pot1-ssDNA, Pot1-Tpz1 and Tpz1-Ccq1 Interactions within fission yeast shelterin complex
Source: PLoS Genet. 2022 Jul 18;18(7):e1010308. doi: 10.1371/journal.pgen.1010308 (PMC9333443; doi:10.1371/journal.pgen.1010308)

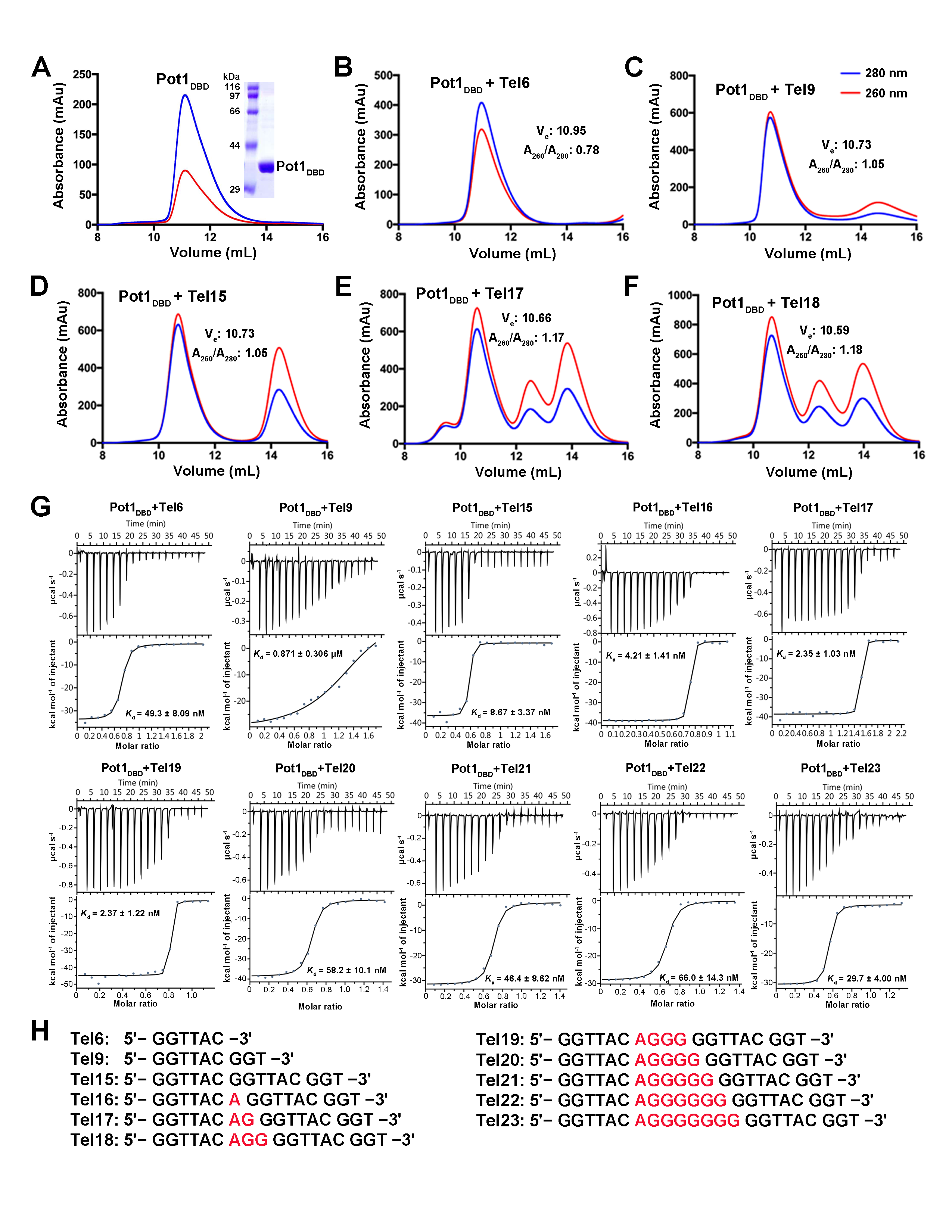

Supplement: S1 Fig — (A) Gel filtration profile of Pot1DBD on a Superdex 200 column. The peak of Pot1DBD was resolved by SDS-PAGE and stained with Coomassie brilliant blue. (B-F) Gel filtration profiles of Pot1DBD binding to different telomeric ssDNAs. The A260/A280 ratios were calculated at the peak elution volume (Ve). (G) ITC measurements of interactions between Pot1DBD and telomeric ssDNAs. (H) Sequences of telomeric repeats used in the biochemical analysis. (TIF) [file pgen.1010308.s001.tif]

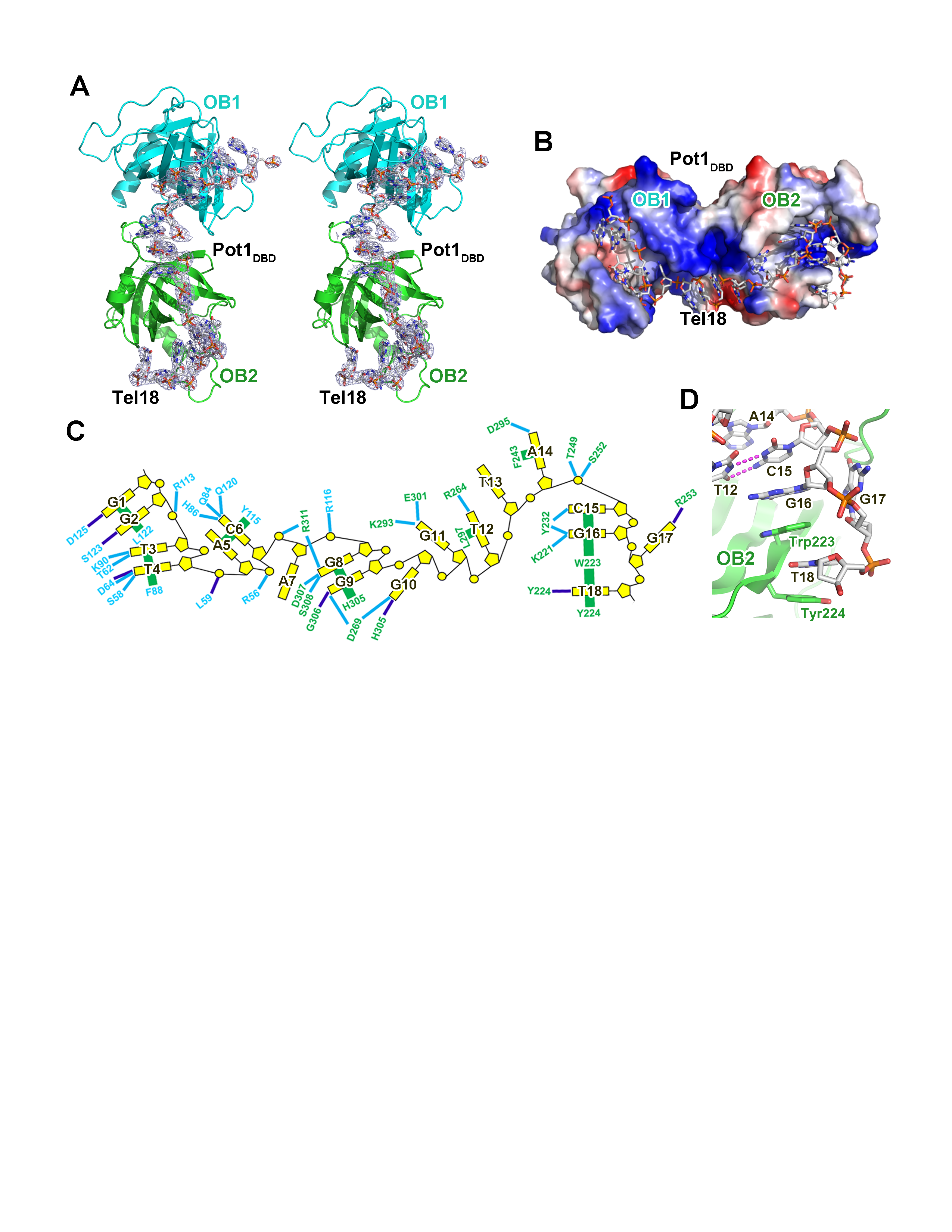

Supplement: S2 Fig — (A) Electron density map of Tel18 in the Pot1DBD-Tel18 complex. Stereo view of the Sigma-A weighted 2Fo-Fc map shows that Tel18 is well ordered in the crystal structure. Refined model of Tel18 is superimposed on the electron density map. Contours are drawn at the 1.0 σ level. (B) Electrostatic potential surface representation of the Pot1DBD protein. Positive potential, blue; negative potential, red (at the 10 kT e-1 level). Tel18 is shown in stick model. (C) Schematic representation of the Pot1DBD-Tel18 interaction. Cyan lines indicate hydrogen bonds and electrostatic interactions between Pot1DBD sidechains and ssDNA phosphates (circles) and bases (rectangles). Green lines indicate van der Waals contacts of Pot1DBD residues with bases as well as the stacking interactions between adjacent bases of Tel18. (D) Well-aligned C15-G16-Trp223-G18-Tyr224 stacking interaction in the Pot1DBD-Tel18 structure. Sidechains of residues important for the interactions are shown in stick models. Dashed magenta lines denote the hydrogen-bonding interactions. (TIF) [file pgen.1010308.s002.tif]

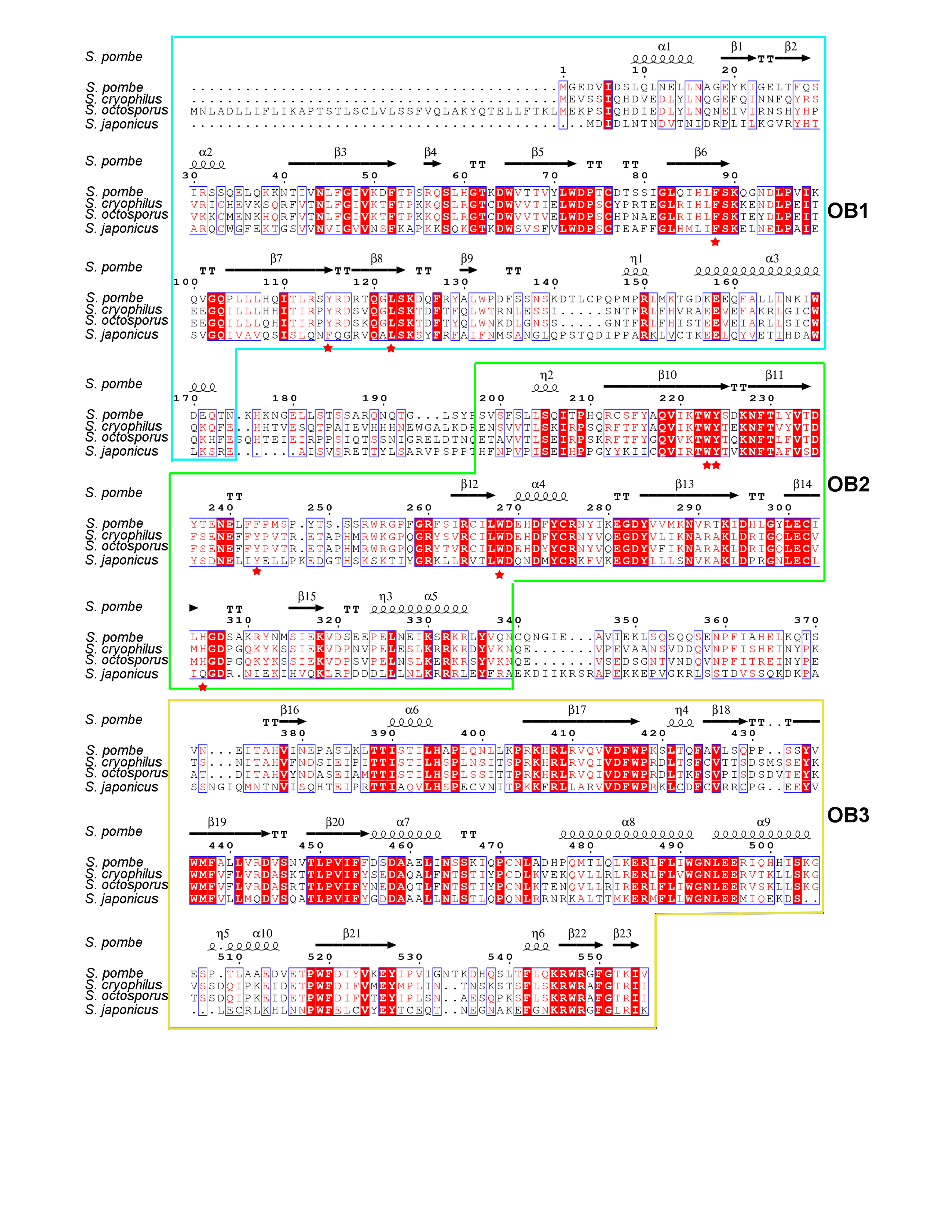

Supplement: S3 Fig — Secondary structure elements of Pot1 are labeled on the top of the sequences. Three OB domains are boxed with respective colors as in Fig 1A. Conserved residues are boxed and highlighted in red. Red stars denote residues important for the stacking interactions observed in the Pot1DBD-Tel18 crystal structure. (TIF) [file pgen.1010308.s003.tif]

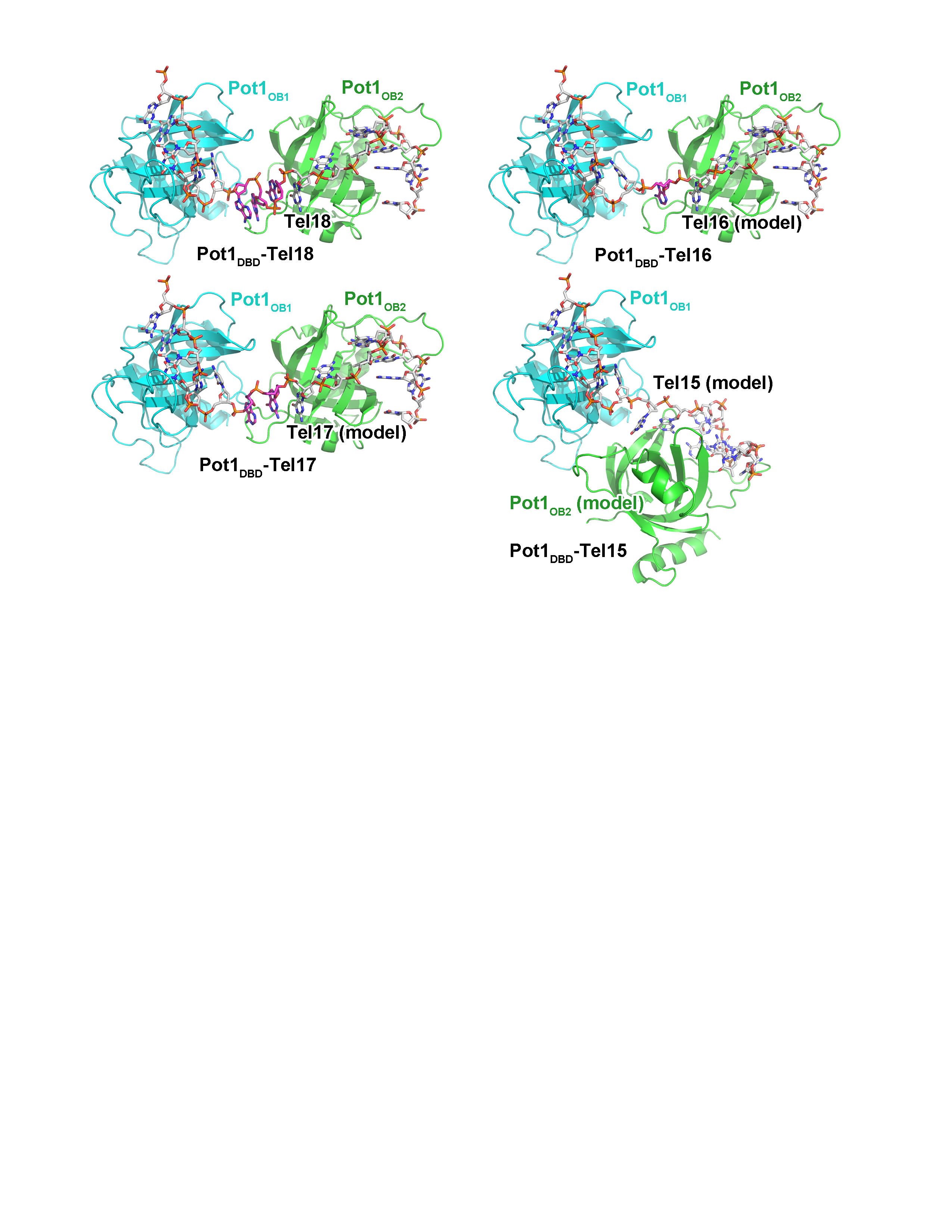

Supplement: S4 Fig — Structural modeling of Pot1DBD bound to two telomeric core repeats with zero (Tel15), one (Tel16) or two (Tel17) linker nucleotides based on the Pot1DBD-Tel18 crystal structure. (TIF) [file pgen.1010308.s004.tif]

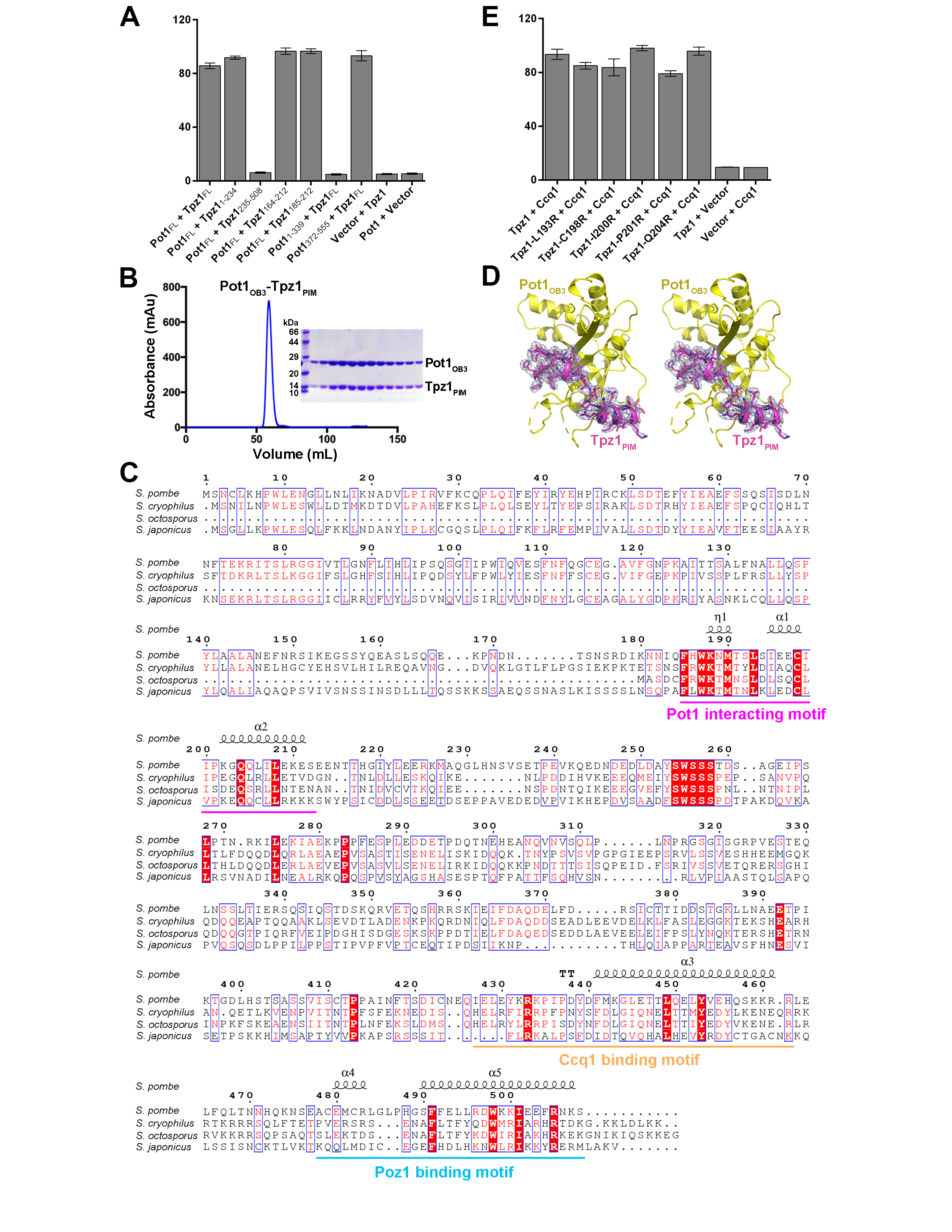

Supplement: S5 Fig — (A) Identification of the domains of Tpz1 and Pot1 that mediate the Pot1-Tpz1 interaction by Y2H analysis. (B) Gel filtration chromatography profile of the Pot1OB3-Tpz1PBM complex. The Pot1OB3-Tpz1PBM complex fractions corresponding to the peak in the gel-filtration profile were resolved by SDS-PAGE and stained with Coomassie brilliant blue. (C) Multiple sequence alignment of Tpz1 proteins from various fission yeast species. Secondary structure elements of Tpz1 are labeled on the top of the sequences. The Pot1-, Ccq1- and Poz1-interacting motifs are indicted. Conserved residues are boxed and highlighted in red. (D) Electron density map of Tpz1PIM in the Pot1OB3-Tpz1PIM complex. Stereo view of the Sigma-A weighted 2Fo-Fc map shows that Tpz1PIM is well ordered in the crystal structure. Refined model of Tpz1PIM is superimposed on the electron density map. Contours are drawn at the 1.0 σ level. (E) Tpz1 mutations that disrupt the Pot1-Tpz1 interaction have no effect on Tpz1-Ccq1 Y2H interactions. (TIF) [file pgen.1010308.s005.tif]

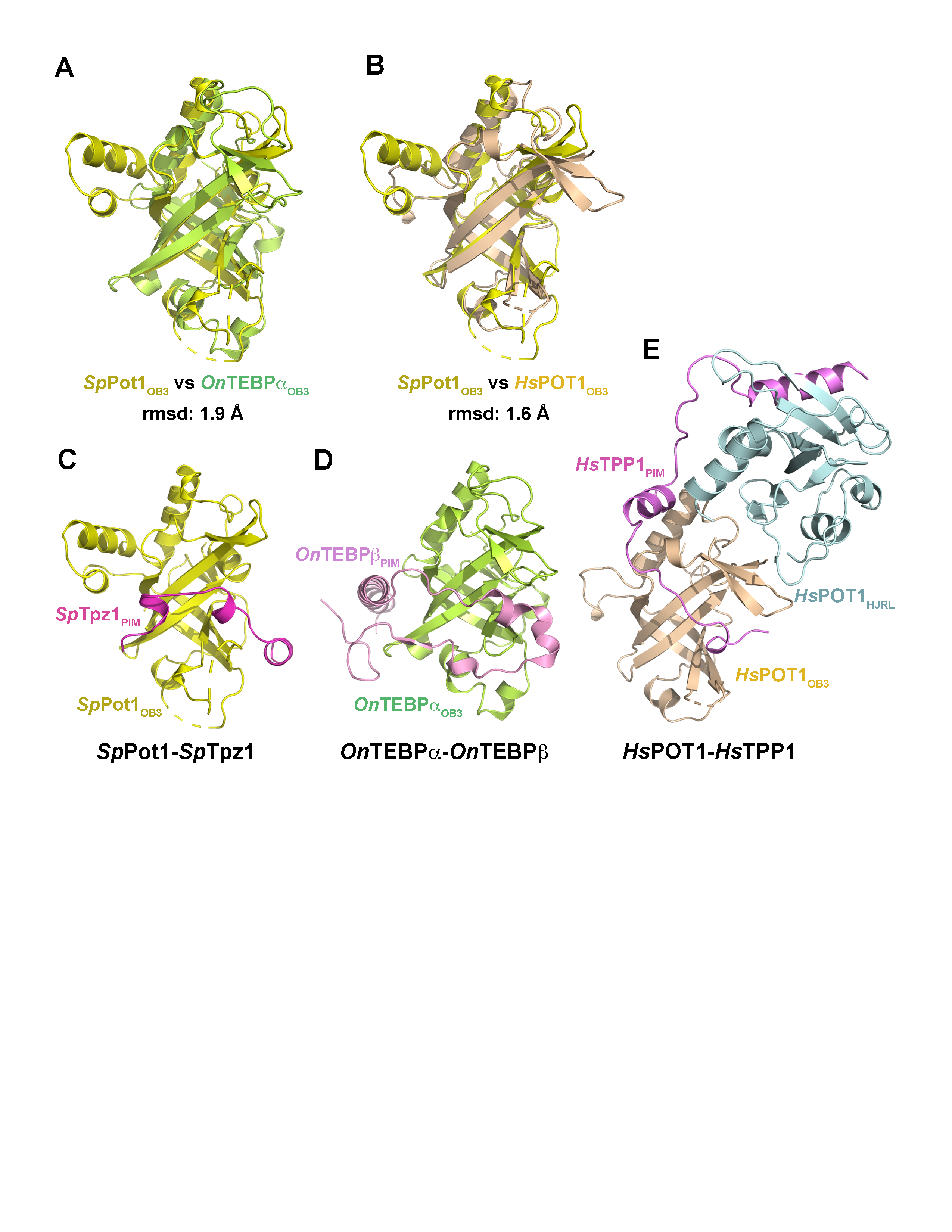

Supplement: S6 Fig — (A) Superposition of S. pombe Pot1OB3 and O. nova TEBPαOB3 crystal structures. (B) Superposition of S. pombe Pot1OB3 and human POT1OB3 crystal structures. (C-E) Structural comparison of the heterodimeric interactions in S. pombe Pot1-Tpz1 (C), O. nova TEBPα-β (D) and human POT1-TPP1 (E) complexes. (TIF) [file pgen.1010308.s006.tif]

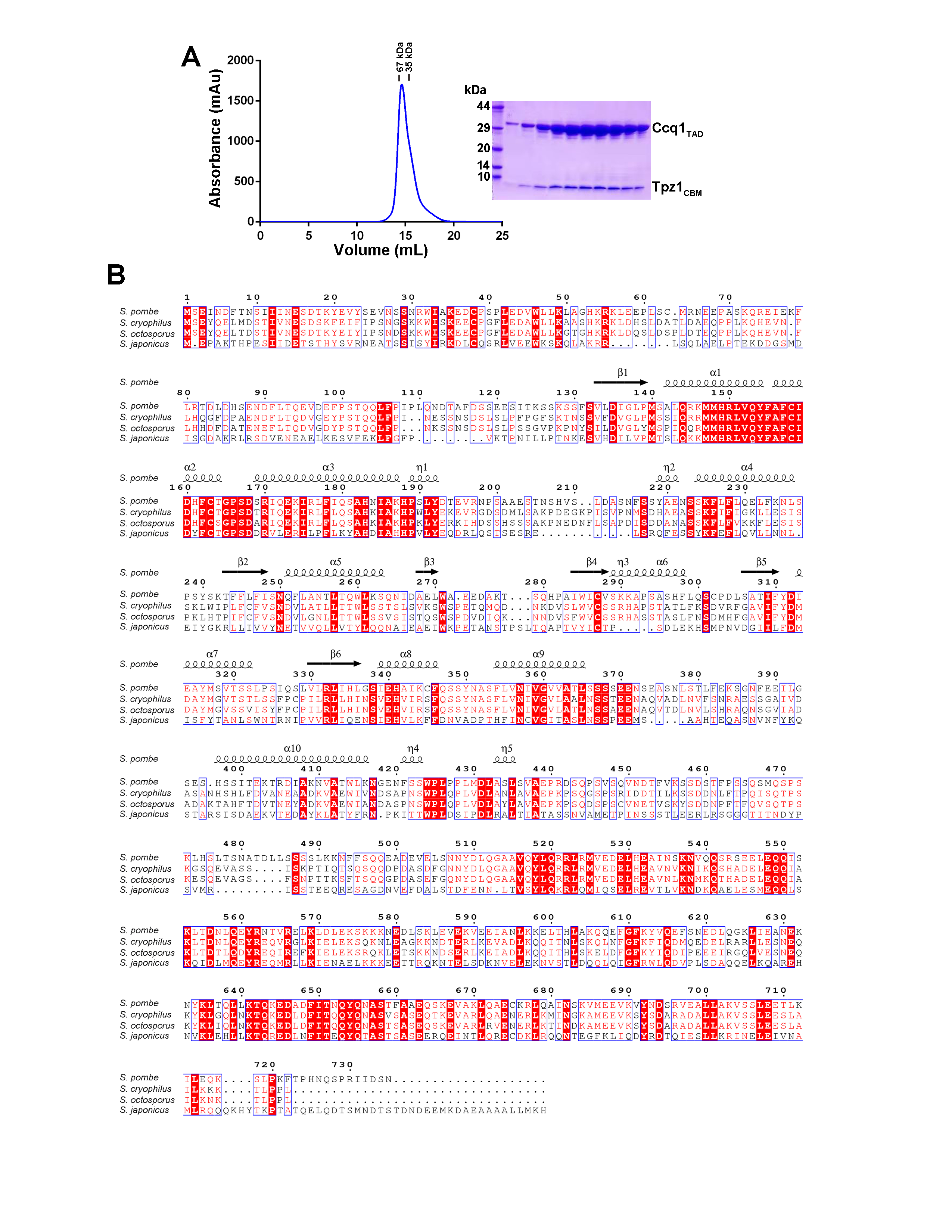

Supplement: S7 Fig — (A) Gel filtration chromatography profile of the Tpz1CBM-Ccq1TAD complex. Elution positions of the 67 and 35 kDa protein markers are indicated. The Tpz1CBM-Ccq1TAD complex fractions corresponding to the peak in the gel-filtration profile were resolved by SDS-PAGE and stained with Coomassie brilliant blue. (B) Multiple sequence alignment of Ccq1 proteins from various fission yeast species. Secondary structure elements of Ccq1 are labeled on the top of the sequences. Conserved residues are boxed and highlighted in red. (TIF) [file pgen.1010308.s007.tif]

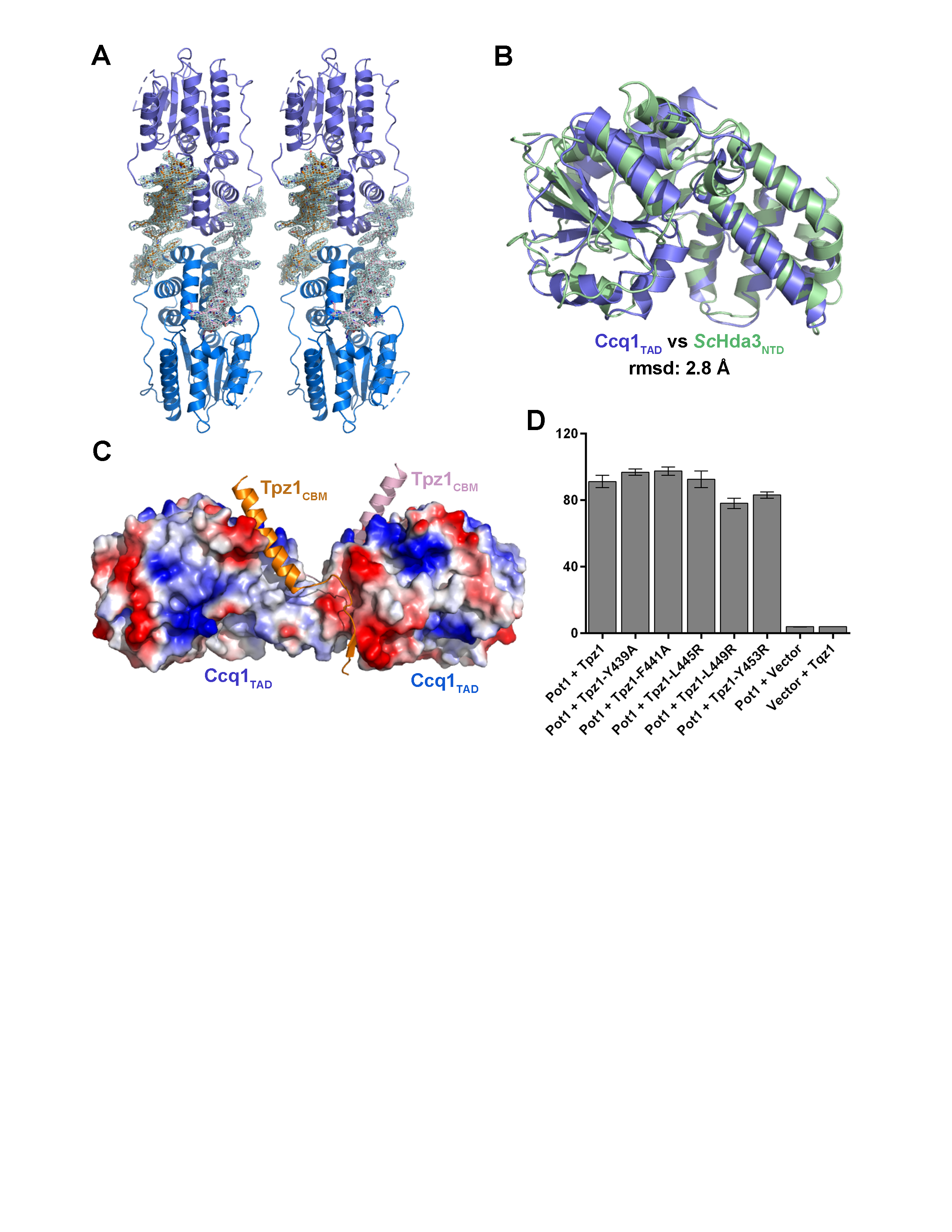

Supplement: S8 Fig — (A) Electron density map of the Tpz1CBM in the Tpz1CBM-Ccq1TAD complex. Stereo view of the Sigma-A weighted 2Fo-Fc map shows that Tpz1CBM is well ordered in the crystal. Refined model of Tpz1CBM is superimposed on the electron density map. Contours are drawn at the 1.0 σ level. (B) Superposition of the Ccq1TAD and ScHda3NTD (PDB: 3HGT) crystal structures. Ccq1TAD and ScHda3NTD are colored in slate blue and palegreen, respectively. (C) Electrostatic surface potential of the Tpz1CBM-binding module on Ccq1TAD (positive potential, blue; negative potential, red). Two Tpz1CBM molecules are in ribbon representation and colored in orange and tint, respectively. (D) Tpz1 mutations that disrupt the Tpz1-Ccq1 interaction have no effect on Pot1-Tpz1 Y2H interactions. (TIF) [file pgen.1010308.s008.tif]

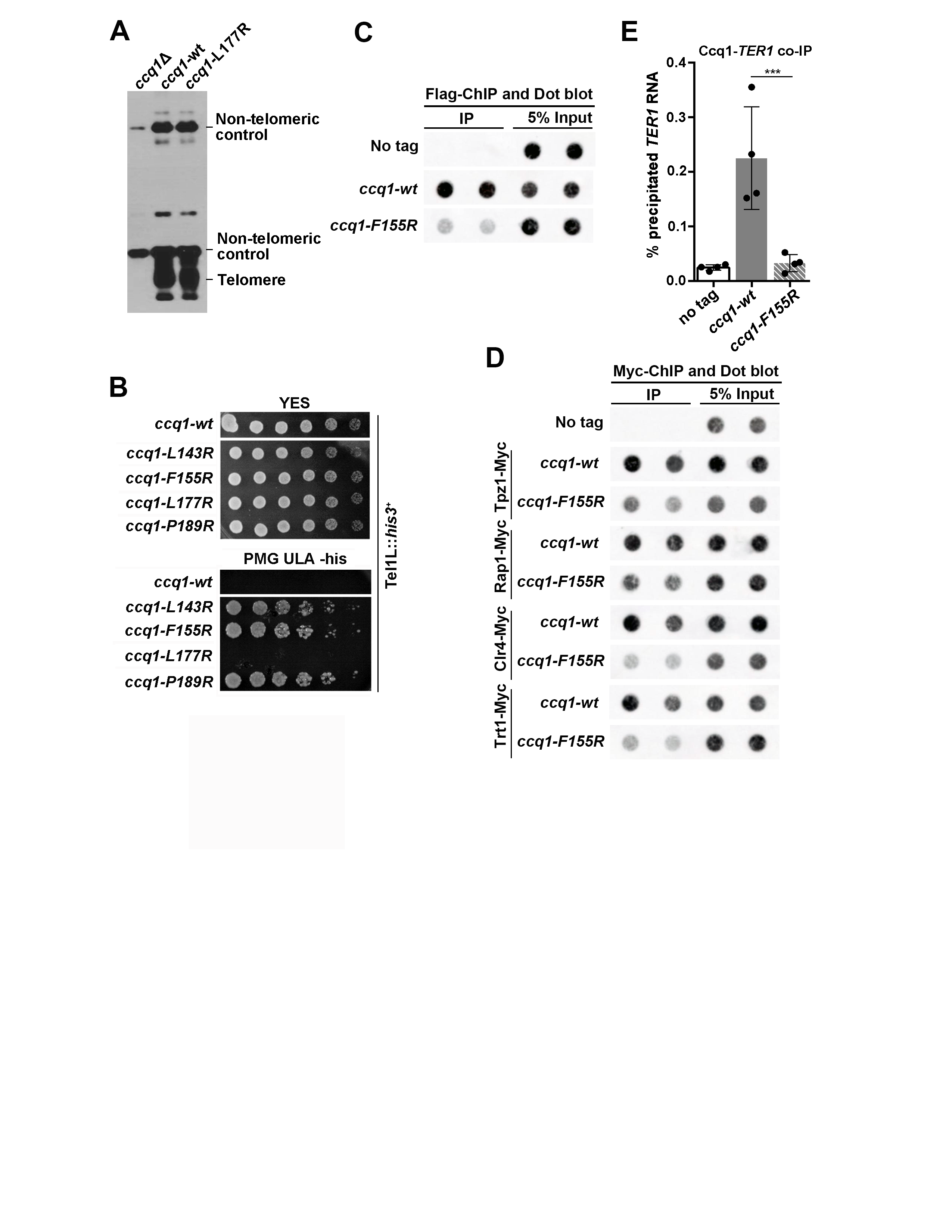

Supplement: S9 Fig — (A) Telomere Southern blot analysis of the negative control ccq1-L177R cells. Genomic DNAs were digested with EcoR I and subjected to Southern blot analysis with a telomere-specific probe. (B) Effects of Ccq1 mutations on the transcriptional silencing of his3+ reporter gene inserted adjacent to the telomeric region. Equal amounts of 10-fold dilution series of cultures were spotted on YES or Pombe Medium Glutamate supplemented with uracil, leucine, and adenine (PMG ULA) (-histidine) plates. (C and D) Effects of the Ccq1-F155R mutation on telomere association for Ccq1, Tpz1, Rap1, Trt1, Clr3 and Clr4 were monitored by dot blot ChIP assays. (E) Co-IP of Ccq1 and TER1 in vivo. Data are represented as mean ± s.e.m. from four independent experiments. (TIF) [file pgen.1010308.s009.tif]

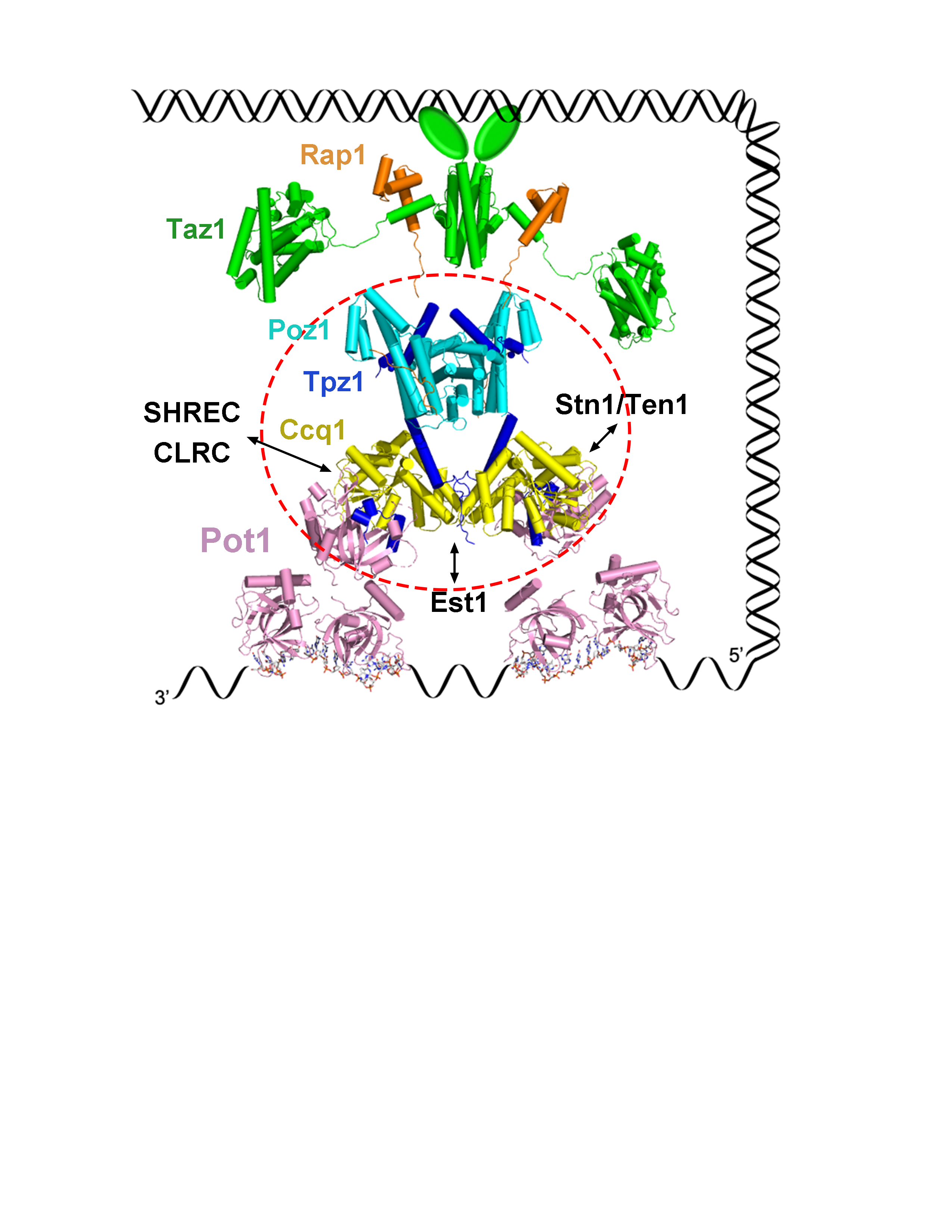

Supplement: S10 Fig — Ribbon diagrams of the shelterin complex based on the atomic structures of Pot1DBD-Tel18, Pot1OB3-Tpz1PIM, Tpz1CBM-Ccq1TAD, Rap1PBM-Poz1-Tpz1PBM (PDB: 5XXF), Rap1RCT-Taz1RBM (PDB: 2L3N) and Taz1DD (PDB: 4ZMK). Taz1 and Pot1 respectively bind to telomeric dsDNA and ssDNA regions via their Myb domains and OB folds, and are bridged by Rap1, Poz1 and Tpz1 via protein-protein interactions. The dimeric state of the shelterin complex is mediated by Taz1DD, Poz1 and the (Tpz1CBM-Ccq1TAD)2 heterotetramer. (TIF) [file pgen.1010308.s010.tif]
