# Supplementary material for: Structural insights into Pot1-ssDNA, Pot1-Tpz1 and Tpz1-Ccq1 Interactions within fission yeast shelterin complex
Source: PLoS Genet. 2022 Jul 18;18(7):e1010308. doi: 10.1371/journal.pgen.1010308 (PMC9333443; doi:10.1371/journal.pgen.1010308)
Supplement: Attachment — Submitted filename: Sun_rebuttal_letter.pdf [file pgen.1010308.s015.pdf]

Point-by-point Responses to Reviewers' Comments

Reviewer #1:

This study describes a series of new crystal structures of multiple protein-protein and protein-DNA interfaces within *S. pombe* shelterin. Specifically, the authors solve the structure of Pot1 DNA-binding domain with a long single stranded telomeric DNA sequence, Pot1-Tpz1 interface, and Tpz1-Ccq1 interface. The new Pot1-DNA structure recapitulates most of the past findings of the DNA-bound structures of individual OB domains while explaining how Pot1 can accommodate spacer sequences between the hexameric telomere repeats at chromosome ends. The Pot1-Tpz1 structure reveals similarities between how this interface is established in various model organisms including humans. The most novel structure described in the study is that of the "butterfly with antenna" shaped Tpz1-Ccq1 complex. The new protein-protein interfaces described in this study are validated using mutants analyzed by methods including yeast two hybrid, immunoprecipitation, telomere length analysis, and telomere ChIP. Overall there is a large amount of high-quality structural data in this manuscript that is backed up by biochemical validation. Although much of the results recapitulate previous findings and predictions based on other homologs, this is still an important contribution to model-system telomere structural biology. Below are major and minor points of critique.

Thanks!

Major points:

1. In multiple parts of the paper, the authors suggest that the Pombe Pot1 DNA binding mode is different from human because the human OB1-OB2 module is locked in one conformation. In a recent paper published in PLOS ONE, the Rhodes group has solved cryo-EM structures of human POT1-TPP1 to suggest that the OB1 and OB2 are not locked into a rigid body but can rather adopt different orientations about each other. Additionally, the same study showed how human POT1 can accommodate non-telomeric sequences between hexameric repeats, just like the *S. pombe* Pot1 protein. Unless the authors have a good reason to rebut these findings, the writing of the manuscript must take into consideration these recent data, which suggest that the human and Pombe OB1-OB2 modules are both flexible.

Thanks for this good point. we agree with this reviewer that the human and *S. pombe* OB1-OB2 modules might be both flexible. Based on analysis of the *S. pombe* $Pot1_{DBD}$-Tel18 structure, we suggest that the structurally separable $Pot1_{OB1}$ and $Pot1_{OB2}$ together with the long flexible loop between them endow *S. pombe* $Pot1_{DBD}$ with more flexibility capable of binding degenerate telomeric sequences. In the revised manuscript, we have taken the recent cryo-EM analysis of human POT1 into account as suggested (Pages 10-11 Lines 203-210).

"A recent cryo-EM analysis has revealed alternative conformations of the two OB folds of human POT1 suggestive of its plasticity in DNA binding. Here, we consider that the structurally separable Pot1$_{OB1}$ and Pot1$_{OB2}$ together with the long flexible loop between them endow *S. pombe* Pot1$_{DBD}$ with more flexibility capable of binding degenerate telomeric sequences. This is also supported by biochemical data that the binding affinity of Pot1 is not significantly affected by addition of spacer sequences (Fig 1B and S1G Fig)."

2. A justification is not provided for the kind of mutation that is made for the various interfaces. Why were Pot1 and Tpz1 residues always mutated to arginine in Fig 3G? Why were some of the Tpz1 mutations in Fig 5G alanine substitutions and other arginine substitutions? An arginine change would be expected to be more drastic, which would agree with the trend in the phenotype with these mutants.

Hydrophobic contacts at the interface are the major driving force underlying the Pot1$_{OB3}$-Tpz1$_{PIM}$ and Tpz1$_{CBM}$-Ccq1$_{TAD}$ interaction. We agree with this reviewer that an arginine substitution is more drastic than an alanine substitution. In order to completely disrupt the hydrophobic contacts, the hydrophobic residues were almost individually substituted with a positively charged arginine residue, that is commonly used in mutational assays (Harland JL…Nakamura TM, PLoS Genetics, 2014, 10:e1004708; Moser BA…Nakamura TM, Mol Biol Cell, 2015, 26:3857-3866; Jun HI…Qiao F, Genes Dev, 2013, 27:1917-31; Liu J…Qiao F, Cell Rep,2015, 12:2169-80). In contrast, the charged residues (Tpz1-K432A, Tpz1-R433A, Tpz1-D438A, Tpz1-H456A) were individually substituted with an alanine residue.

3. In the yeast two hybrid (Y2H) assay in Fig. 3G, there is no evidence that the mutant protein is expressed or is folded? At least for the Tpz1 mutants, the authors could perform a Y2H against Ccq1 to show that the mutants are active in this biochemical function. This would provide strong evidence that the mutants are produced and are stably folded. In fact in Fig. 5H it seems like the two tested mutants are not expressed well. What is the expression level of the other mutants used in the Y2H in Fig. 5G? Y2H of these Tpz1 mutants with Pot1 should be performed to confirm that the mutants can still bind Pot1 as the mutations shouldn't affect this interface.

Thanks for this good point. Following this reviewer's suggestion, we have added the relevant Y2H experiments in the revised manuscript (Pages 13 Lines 263-264; Page 19 Lines 376-377; the revised S5E and S8D Fig).

4. Although the differences look large for the functional data in the manuscript, no statistical significance is provided. P-values should be reported for all effects that the authors propose are significant.

We have made such a change accordingly in the revised Fig 4C-4I.

5. Figure 2 is largely based on modeling of shorter DNA substrates. However, the method used for modeling was not described. This must be described in more detail, elaborating on whether energy minimization and/or geometry optimization was used in the protocol. Along the same line, the geometry (bond angles, bond lengths, dihedral angles) of the modeled nucleotides/linkages must be detailed along with how much they deviate from the standard values. While it is understandable that the authors did not solve new structures with each shorter DNA substrate, the modeling data should only be shown in the main figure if a rigorous computational method was used to generate the models. Otherwise, the modeling should be moved to the supplement. This is especially relevant to Tel15-bound structure, where the authors suggest a swinging away of the OB domains from each other.

Thanks for this good point. Based on the $Pot1_{DBD}$-Tel18 structure, we generated the $Pot1_{DBD}$-ssDNA structural models with 0-3 nucleotide linkers. Although energy minimization was not applied in the modeling, the structural models were generated based on tight conformational and stereochemical constraints on both DNAs and proteins. In the revised manuscript, following this reviewer's suggestion, we have described this structural modelling in more details (Pages 9 lines 181-184), and moved the panel of the modeling data from original Fig 2 to revised supplementary Figure S4 (revised S4 Fig).

6. Figure 3 is already dedicated to the Pot1-Tpz1 structure. It doesn't seem justified to show superpositions of the same structure on other homologous structures as a new figure (figure 4). It seems most appropriate to move Figure 4 into the supplement or merge it with other panels in figure 3.

Following this reviewer's suggestion, we have moved it into the supplementary (the revised S6 Fig).

7. The authors make a point about differences in nt # 11, 12 and 13 between their structure and the OB2-9mer structure. What is the significance of this difference? Are the authors suggesting these are two alternative conformations or are they saying one is correct and the other isn't? In either case, what is the physiological importance of attaining one conformation versus the other? Can the authors do additional experiments to determine which conformation is important for DNA binding affinity or dynamics or telomere function in vivo?

Structures of $Pot1_{OB2}$ with different ssDNA ligands reveal multiple binding modes of $Pot1_{OB2}$ that explain its nonspecific recognition of ssDNA (Dickey TH…Wuttke DS, Structure, 2013, 21: 121-132). In addition, $Pot1_{OB2}$ confers only moderate sequence specificity for nucleotides G11, G16 and T18 (Croy JE…Wuttke DS, Biochemistry, 2009, 48: 6864-75). Thus, the $Pot1_{OB2}$-Tel9 complex might exhibit dynamic conformations. The difference from nucleotides T12 and T13 in the $Pot1_{DBD}$-Tel18 and $Pot1_{OB2}$-Tel9 complexes could be due to the conformational dynamics of the

$Pot1_{OB2}$-Tel9 complex. Although we are currently unable to discriminate the two conformations for DNA binding affinity experiments, our biochemical data demonstrated that $Pot1_{DBD}$-Tel18 has a greater binding affinity than two subdomains, especially than $Pot1_{OB2}$-Tel9 (S1G Fig), suggesting that our $Pot1_{DBD}$-Tel18 structure likely reflects the conformation of $Pot1_{DBD}$ binding to two telomeric core repeats with spacers.

8. In general, the novelty of the Pot1-DNA structure is not described precisely. There are already two structures of Pot1 domains bound to DNA. Throughout the manuscript (including abstract, author summary, and multiple instances in the main text) it must be specified that the novelty of the structure presented here is the presence of both OB domains, and more importantly, the presence of the spacer sequences between hexameric repeats.

Thanks for pointing out this good issue. We have specified the novelty of our $Pot1_{DBD}$-ssDNA structure accordingly.

In Page 2 Lines 31-34, "While individual structures of the two DNA-binding OB folds of Pot1 are available, structural insight into recognition of telomeric repeats with spacers by the complete DNA-binding domain ($Pot1_{DBD}$) remains an open question."

In Page 3 Lines 57-59, "Although individual OB-fold subdomains structures have been characterized, structural information about into the complete $Pot1_{DBD}$ bound to telomeric repeats with spacers remains to be revealed."

In Page 6 Lines 109-119, "Notably, the *S. pombe* telomeric sequence is irregular, in which the 5'-GGTTAC-3' core sequence are separated by 0-8 linker nucleotides. Structural information about the complete $Pot1_{DBD}$ bound to telomeric repeats with spacer sequences still has yet to be revealed, hindering our understanding of how *S. pombe* Pot1 recognizes the irregular cognate telomeric sequence."

9. In multiple places in the manuscript (including abstract and main text), the authors seem to suggest that this study shows the structural basis of the heterochromatin function of Ccq1. However, this structural study does not have heterochromatin components like SHREC. The studies performed here show that the loss of the Tpz1-Ccq1 results in a loss of Ccq1 from telomeres, which results in heterochromatin defects. Thus the study is not showing how Ccq1 is performing its heterochromatin function. Instead it is showing that Ccq1 must be recruited to telomeres to perform its functions. This must be clarified and any overstatements of the facts revised.

Following this reviewer' suggestion, we have lowered the tune about the statement of "structural basis of the heterochromatin function of Ccq1", and rephrased our statements in the revised manuscript as the following.

In Page 2 Lines 34-37, "Moreover, structural information about the Tpz1-Ccq1 interaction requires to be revealed for understanding how the specific component Ccq1 of *S. pombe* shelterin is recruited to telomeres to function as an interacting hub."

In Page 2 Lines 41-43, "Our analyses of Tpz1-Ccq1 reveal the structural basis for the essential role of the Tpz1-Ccq1 interaction in telomere recruitment of Ccq1 that is required for telomere maintenance and telomeric heterochromatin formation."

In Page 3 Lines 62-64, "…but also for the essential function of the Tpz1-Ccq1 interaction in Ccq1 recruitment to telomeres for telomere maintenance and telomeric heterochromatin formation."

In Page 6 Lines 122-124, "…the essential function of the Tpz1-Ccq1 interaction in Ccq1-dependent telomere maintenance and telomeric heterochromatin formation."

In Page 22 Lines 428-430, "Taken together, we conclude that the Tpz1-Ccq1 interaction plays an essential role in Ccq1 recruitment to telomeres that functions as a platform for telomerase and heterochromatic complexes SHREC and CLRC, …"

In Page 25 Lines 488-490, "After telomeric G-strand replication, Ccq1 recruits the SHREC and the CLRC complexes to telomeres to establish telomeric heterochromatin, …"

Minor points:

1. Remove the word "While" at the beginning of the sentence in line 108; start directly with "Structures…"

Corrected.

2. In line 215, Asp415 is mentioned as part of a hydrophobic pocket. Either the term hydrophobic should be removed or toned down (mostly or largely hydrophobic) or Asp415 should not be listed there.

The residue Asp415 have been removed.

3. In line 31, it is most accurate to mention "DNA-bound" structures of POT1 domains.

Corrected.

4. In line 199: change to "Similar to how human POT1 interacts with TPP1"

Corrected.

5. In line 421 “constrain” should be “constraint”

Corrected.

6. In line 436 Figure 7 human shelterin diagram is not consistent with the current understanding of the stoichiometry, which has TRF2:RAP1 = 2:2.

Corrected in the revised Fig 5.

Reviewer #2:
In this ms by Sun et al, the authors determined 3 crystal structures, Pot1(DBD)-ss telomeric DNA, Pot1(OB3)-Tpz1(PIM), and Tpz1(CBM)-Ccq1(TAD). These are 3 individual interfaces in the telomere nucleoprotein complex, different from the comprehensive structure of the whole trimer as claimed by the authors in the title, “Telomeric Overhang Binding Pot1-Tpz1-Ccq1 Complex”.

Thanks for this reviewer’s comments and suggestions, which help us improve our manuscript significantly.

Pot1(DBD)-ss telomeric DNA is not entirely new because the structures of Pot1(OB1)-ssDNA and Pot1(OB2)-ssDNA structures were solved a while ago with Pot1(OB1)-ssDNA in 2003. In addition, the mechanism by which degenerate ss telomeric DNA seq is recognized was extensively elucidated by a serial of biochemical and biophysical work from the Baumann and Wuttke labs more than a decade ago.

Although individual OB-fold subdomains structures have been characterized (Lei M…Cech TR, Nature, 2003, 426: 198-203; Dickey TH…Wuttke DS, Structure, 2013, 21: 121-132), structural information about the complete Pot1$_{DBD}$ bound to telomeric repeats with spacers remains to be revealed. Pot1$_{DBD}$ bound to telomeric repeats has long been considered to be difficult to crystallization (Dickey and Wuttke DS, Nucleic Acid Res, 42: 9656-9665). Here, we reported the crystal structure of the complete Pot1$_{DBD}$ in complex with two telomeric repeats with three linker nucleotides (Pot1$_{DBD}$-GGTTACAGGGGTTACGGT, Pot1DBD-Tel18). This novel structure, when combined with biochemical data from the Baumann and Wuttke labs, provides structural basis for *S. pombe* Pot1 recognition with the irregular cognate telomeric sequence (this point is also stated by the reviewer 1).

For the Pot1(OB3)-Tpz1(PIM) structure, its homologs in humans and in Ciliates have been solved and the work here presents little new structural insight.

The *S. pombe* Pot1-Tpz1 complex showed an overall structural similarity with human POT1-TPP1 and *O. nova* TEBPα-β complexes. Despite these similarities, how the OB folds recognize their partners display some unique features in the three complexes,

inferring that variations likely have evolved to meet the special functional need in different organisms (this point is also stated by the reviewer 3).

Moreover, the highly flexible nature of *S. pombe* shelterin complex has greatly impeded our structural characterization of this important complex in fission yeast. In previous studies, we and others have determined the crystal structures of the Poz1-Tpz1-Rap1, Taz1-Rap1 subcomplexes and various domains of Taz1 and Pot1. The crystal structures of three additional modules reported in this study, when combined with previous structures, enable us to build an atomic model for the entire *S. pombe* shelterin complex.

Tpz1(CBM)-Ccq1(TAD) is a new structure; however, the claimed new "functional insights into Ccq1-dependent telomere maintenance and telomeric heterochromatin formation" is merely repeat of previously published results using a slightly different mutants disrupting the same interface (as explained below).

Thanks for this good point. Although the roles of the Tpz1-Ccq1 interaction in telomere maintenance and telomeric heterochromatin formation have been widely characterized, our novel $Tpz1_{CBM}$-$Ccq1_{TAD}$ structure provides structural basis for the essential role of the Tpz1-Ccq1 interaction in telomere recruitment of Ccq1 that is required for telomere maintenance and telomeric heterochromatin formation. Our functional data based on the structure-derived mutants functionally verified the Tpz1-Ccq1 interface structure. Thus, our structural and functional analysis of the Tpz1-Ccq1 interaction, when combined with previous functional data, provide structural basis for understanding how the specific component Ccq1 of *S. pombe* shelterin is recruited to telomeres functioning as an interacting hub.

In the revised manuscript, we have lowered the tune about the statement of "functional insights into Ccq1-dependent telomere maintenance and telomeric heterochromatin formation", and rephrased it into "Our analyses of Tpz1-Ccq1 reveal structural basis for the essential role of the Tpz1-Ccq1 interaction in telomere recruitment of Ccq1 that is required for telomere maintenance and telomeric heterochromatin formation." (In Page 2 Lines 34-37; In Page 2 Lines 41-43; In Page 3 Lines 62-64; In Page 6 Lines 122-124; In Page 22 Lines 428-430; In Page 25 Lines 488-490).

Overall, this paper provides useful "for the record" structures of the fission yeast shelterin components, but significantly overclaims its new biological insight. It is a solid candidate for journals publishing protein structures (such as, Acta D), but lacks the level of biological insight and rigor in functional analysis for PLOS Genetics.

The highly flexible nature of *S. pombe* shelterin complex leads this important complex to be recalcitrant to cryo-EM determination. Our three crystal structures, when integrated with previous structures determined by us and others, enable us to

build an atomic model for the entire *S. pombe* shelterin complex. Thus, as also stated by the reviewer 1, our study is an important contribution to model-system telomere structural biology. Moreover, as also stated by the reviewer 3, our study helps to understand how the telomere is regulated by the shelterin complex in higher eukaryotes.

Thus, we believe that this article will certainly be appreciated by the greater field of telomere biology and the broad readership of PLoS Genetics.

Major points:

1. Title "Structural Insights into Fission Yeast Telomeric Overhang Binding Pot1-Tpz1-Ccq1 Complex" misleads readers to think the paper solved the whole Pot1-Tpz1-Ccq1 complex structure. Need to change it to "Crystal structures of Pot1(DBD)-ss telomeric DNA, Pot1(OB3)-Tpz1(PIM), and Tpz1(CBM)-Ccq1(TAD)".

Thanks for pointing out this good issue. We have modified the title to "Structural insights into Pot1-ssDNA, Pot1-Tpz1 and Tpz1-Ccq1 interactions within fission yeast shelterin complex".

2. "In this study, we determine the crystal structures of the Pot1-ssDNA, Pot1-Tpz1, and Tpz1-Ccq1 subcomplexes, providing not only structural basis for the telomeric overhang-binding module Pot1-Tpz1-Ccq1…" The authors must specify the residue range for each protein in the structures. Otherwise, it misleads the readers to think it is the full-length protein.

Thanks for this good point, and we have specified the residue range for each protein in our structures (Page 2 Line 38; Page 3 Line 60; Page 6 Lines 120-121).

Moreover, for "telomeric overhang-binding module Pot1-Tpz1-Ccq1", it has no biological foundation to include Ccq1, because there is no evidence that Ccq1 works with Pot1-Tpz1 ssDNA binder. In fact, Ccq1 has more functional relationship to Poz1 because double deletion of poz1 and ccq1 causes telomere deprotection.

We agree with this reviewer, and accordingly we have modified the statement on "telomeric overhang-binding module Pot1-Tpz1-Ccq1" throughout the revised manuscript as follows.

Title: the title has been rewritten to "Structural insights into Pot1-ssDNA, Pot1-Tpz1 and Tpz1-Ccq1 interactions within fission yeast shelterin complex".

In Page 2 Lines 29-31, "…shelterin consists of telomeric single- and double-stranded DNA-binding modules Pot1-Tpz1 and Taz1-Rap1 connected by Poz1, and a specific component Ccq1".

In Page 2 Lines 43-45, "our findings provide valuable structural information regarding interactions within fission yeast shelterin complex at 3' ss telomeric overhang".

3. Pot1-Tpz1 is the complete ss telomeric DNA binder, not Pot1 by itself. Cech lab showed that Pot1-Tpz1 binds to ssDNA 10 times stronger than Pot1 by itself, indicating the contribution from Tpz1 to ssDNA binding just as ciliate TEBP-b does. The structural and biochemical study would generate new and complete picture of shelterin-ssDNA binding only if Pot1-Tpz1 complex is employed as the whole entity. To satisfy the authors' claim of "telomeric overhang-binding module", full length Pot1-Tpz1 complex needs to be characterized here structurally.

Following this reviewer's suggestion, we have tried to reconstitute the Pot1-Tpz1 complex bound to ssDNA. First, we co-expressed full-length Pot1 and Tpz1, but unfortunately we were unable to obtain a stable full-length Pot1-Tpz1 complex. Thus, we co-expressed and purified full-length Pot1 and $Tpz1_{PIM}$ in *Escherichia coli* with His_6-SUMO and GST tags. After mixing with Tel18, we obtained a stable $Pot1_{FL}$-$Tpz1_{PIM}$-Tel18 complex (Reviewer Figure 1), but this complex remained recalcitrant to crystallization. Due to these technical issues, we are currently unable to structurally characterize the full-length Pot1-Tpz1 complex bound to telomeric ssDNA. Next, we will try to determine the structure of the entire *S. pombe* shelterin complex using cryo-EM method.

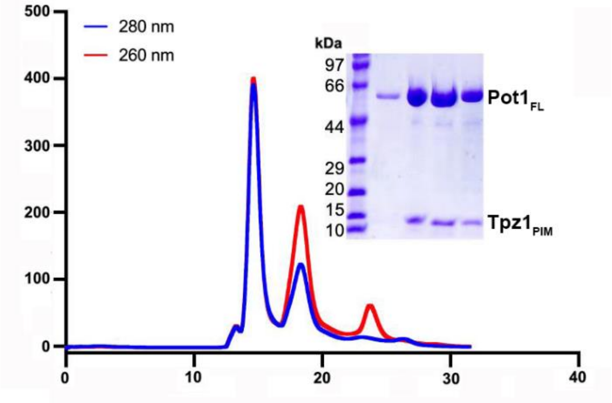

Reviewer Figure 1. Gel filtration profile of the $Pot1_{FL}$-$Tpz1_{PIM}$-Tel18 complex on a Superdex 200 column. The fractions of the $Pot1_{FL}$-$Tpz1_{PIM}$-Tel18 complex were resolved by SDS-PAGE and stained with Coomassie brilliant blue.

Thus, we have modified the statement on "telomeric overhang-binding module Pot1-Tpz1-Ccq1" as suggested by this reviewer.

Title: the title has been rewritten to "Structural insights into Pot1-ssDNA, Pot1-Tpz1 and Tpz1-Ccq1 interactions within fission yeast shelterin complex".

In Page 2 Lines 29-31, "…shelterin consists of telomeric single- and double-stranded DNA-binding modules Pot1-Tpz1 and Taz1-Rap1 connected by Poz1, and a specific component Ccq1".

4. "Our biochemical analysis using purified proteins showed that a short and highly conserved fragment of Tpz1 (residues 185-212) is sufficient to maintain a stable interaction with Pot1-372-555". The biochemical assay here (co-migration in gel filtration) only shows binding, but other parts of Tpz1 or Pot1 involved in the interaction might be omitted. To ensure that Tpz1-185-212 and Pot1-372-555 are the minimum but comprehensive interaction units for Pot1-Tpz1 interaction. The authors need to show that Tpz1-185-212 and Pot1-372-555 interaction has the same affinity as the full-length Pot1 and Tpz1.

Following this reviewer's suggestion, we have added the Y2H assay to confirm the domains of Pot1 and Tpz1 that mediate the Pot1-Tpz1 interaction (the revised S5A Fig).

5. The fission yeast molecular biology data (Fig 6) are merely repeats of similar data (disrupting the same interface using either the same or different mutants) and the data quality is poor. Barely any new biological insight was revealed from this figure, in contrast to what the authors claimed.

In this revised manuscript, we have lowered the tune and rephrased the statement as "Our analyses of Tpz1-Ccq1 reveal structural basis for the essential role of the Tpz1-Ccq1 interaction in telomere recruitment of Ccq1 that is required for telomere maintenance and telomeric heterochromatin formation." (Page 2 Lines 41-43).

Our functional experiments (the revised Fig 4) were performed according to the published papers (Harland JL…Nakamura TM, PLoS Genetics, 2014, 10: e1004708; Moser BA…Nakamura TM, Mol Biol Cell, 2015, 26: 3857-3866), and we believe that the data quality is comparable to the published data.

a. There is no molecular weight ladder, no labeling indicating which band represents telomeres. What is the identity of the top band? loading control? if so, which gene? Some other gene needs to be used for this purpose because it is too close to the telomere band. What is the difference between the two lanes of the same genetic background?

First, we have added the molecular weight ladder in the revised Fig 4A. The telomere Southern blot was performed according to the published paper by Nakamura lab, and the data quality is comparable to their published data (Harland JL…Nakamura TM,

PLoS Genetics, 2014, 10: e1004708; Moser BA…Nakamura TM, Mol Biol Cell, 2015, 26: 3857-3866). The top band represents non-telomeric control served as a relative-mobility control.

Second, the haploid *ccq1* mutant strains were obtained by direct transformation with mutated DNA fragments with kanMx6 (kan^r) and confirmed by PCR. The correct mutant strains were re-streaked three successive times on YES plates for telomere determination, that might be the reason for the slight difference in telomere maintenance between two strain even with the same genetic background.

b. Based on the data in Fig. 5b, the degree of telomeric silencing does not correlate with the degree of Tpz1-Ccq1 disruption. For example, Ccq1-M147R mutant disrupts Tpz1-Ccq1 interaction as effective as L151R; however, M147R is almost at the WT level of silencing. In case like this, H3K9-CHIP, Western blot of major proteins involved (Tpz1, Ccq1, and Clr4) are required to sort things out.

In our original submission, we did not include any data of the Ccq1-M147R mutant in the functional analysis of telomere length maintenance and heterochromatin formation (original Fig 6A and 6B).

c. The authors use Ccq1-TER1 RNA co-IP as a test to evaluate the role of Ccq1-Tpz1 interaction in telomerase recruitment. Due to complex recruitment pathway of telomerase to telomeres, the field standard is to use Trt1-CHIP to evaluate Trt1 recruitment in different genetic background.

Thanks for this good point. Following this review's suggestion, we have added the ChIP experiment for Trt1-13myc (the revised Fig 4G), and the Ccq1-TER1 RNA co-IP result has been moved to supplementary S9E Fig.

d. The authors mentioned, "Notably, the amounts of telomeres associated Tpz1 and Rap1 were also decreased in the ccq1L151R and ccq1F155R cells (Fig. 5e, f), likely due to the telomere loss after the disruption of the Tpz1-Ccq1 interaction". Telomere loss is normalized in the CHIP assay. The fact that the authors observed less telomere association of Tpz1 and Rap1 indicate that the telomere sequence is changed in the ccq1 mutant background. As Cooper lab (Tomita et al G&D 2008) showed that the cell maintains telomeres using the rad51-dependent recombination mechanism. Therefore, the seq composition of the telomeres is different from the telomerase-dependent mode. Slot-blot (instead of qPCR) must be used for the CHIP in this study (Fig 5d,e, f, h, i).

We agree with this reviewer that the *ccq1Δ* mutation results in gradual telomere shorting, and later *ccq1Δ* cells use the rad51-dependent recombination mechanism till the extremely short telomeres (Tomita K and Cooper J P, Genes Dev, 2008, 22:3461-3474).

Following this reviewer' suggestion, we have added the dot blot assay for our ChIP results (the revised S9C and S9D Fig). In this study, early *ccq1* mutants that remain unchanged in the seq composition of the telomeres, were used for ChIP assays. Thus, in the revised manuscript, the ChIP-qPCR results are kept in main text (the revised Fig 4D-4I), and the dot blot ChIP data in supplementary (the revised S9C and S9D Fig).

Minor points:
The authors need to give credit to other labs in the field by discussing the structures and results in the context of what was known. Specific areas include Pot1-Tpz1 and ssDNA binding work from the Wuttke, Cech, and Baumann labs, and Ccq1-Tpz1 interaction work from Nakamura and Jia labs.

We appreciate structural and functional contributions to the *S. pombe* shelterin complex by fellow scientists, such as Wuttke, Cech, Baumann, Nakamura, Qiao and Jia labs. Following this review's suggestion, we have given credit to the above labs when discussing the structures and results throughout the revised manuscript.

Introduction: In the paragraph 2 and 3, we have provided a detailed introduction on structural and functional studies on the shelterin complex; almost published work has been concluded and cited.

Results: First, we discussed our $Pot1_{DBD}$-Tel18 structure in the context of the published $Pot1_{OB1}$-Tel6 and $Pot1_{OB2}$-Tel9 structures (Lei M…Cech TR, Nature, 2003, 426: 198-203; Dickey TH…Wuttke DS, Structure, 2013, 21: 121-132; Smith EW…Rhodes D, PLoS One, 2022, 17(2): e0264073), as well as the biochemical and biophysical work on how Pot1 binds telomeric repeats from the Cech, Baumann and Wuttke labs (Nandakumar J and Cech TR, Nucleic Acids Res, 2012, 40: 235-244; Trujillo KM…Baumann P, J Biol Chem, 2005, 280: 9119-9128; Hiraoka Y…Blackburn EH, Trends Biochem Sci. 1998, 23: 126; Croy JE…Wuttke DS, Biochemistry, 2009, 48: 6864-6875; Altschuler SE…Wuttke DS, Biochemistry, 2011, 50: 7503-7513; Croy JE…Wuttke DS, J Mol Biol, 2006, 361: 80-93).

Second, the published structural and functional studies on the Pot1-Tpz1 and Tpz1-Ccq1 interactions by Nakamura, Qiao and Jia labs were also taken into account when discussing the structures and results of the Pot1-Tpz1 and Tpz1-Ccq1 interactions within *S. pombe* shelterin complex (Chen C…Lei M, Nat Commun, 2017, 8:14929; Rice C…Skordalakes E, Nat Commun, 2017, 8:14928; Harland JL…Nakamura TM, PLoS Genetics, 2014, 10:e1004708; Moser BA…Nakamura TM, Mol Biol Cell, 2015, 26:3857-3866; Jun HI…Qiao F, Genes Dev, 2013, 27:1917-31; Liu J…Qiao F, Cell Rep,2015, 12:2169-80; Wang J…Jia S, Genes Dev, 2016, 30:827-39).

Discussion: In this section, we combined with previous studies, providing an integrated picture for telomere maintenance, telomere protection and telomeric heterochromatin formation in fission yeast. Almost published work has been taken into account.

Reviewer #3:

1) General comments

In this manuscript, Sun et al. have provided structural basis for the recognition of *S. pombe* degenerate telomeric sequences by Pot1 and the essential function of Ccq1 in telomere maintenance and telomeric heterochromatin formation by the determination of the crystal structures of the Pot1-ssDNA, Pot1-Tpz1 and Tpz1-Ccq1 subcomplexes. By this work, the authors are proposing an integrated model depicting how the *S. pombe* shelterin complex assembles and plays its roles at telomere. These findings are suggesting that the shelterin complex has evolved distinct molecular architectures to accommodate different functions in fission yeast and mammals during evolution. This study will help us to understand how the telomere is regulated by the shelterin complex in higher eukaryotes. Most of the conclusions drawn by the authors are supported or suggested by the experimental data. The following points should be modified or answered:

Thanks!

2) Specific comments

Major points:

1. There is no interpretation for the results of Tpz1-W187R, Tpz1-N189R, and Tpz1-M190R mutants in Y2H in Fig. 3G.

Thanks for this good point, we have added the interpretation in the revised manuscript (Page 13 Lines 257-260).

"Individual arginine substitution of Tpz1-Trp187, Tpz1-Asn189 and Tpz1-Met190 showed no effect on the Pot1$_{OB3}$-Tpz1$_{PIM}$ interaction, suggestive of little contribution of the N-terminal of Tpz1$_{PIM}$ to Pot1$_{OB3}$ interaction."

2. ccq1-F177R mutant had better be included as a control in the analyses of Fig. 6A and B (or in supplemental Figures), because Ccq1-F177R mutation does not disturb Tpz1-Ccq1 interaction.

Thanks for pointing out this good issue, and we have added the according experiments (Page 20 Lines 400-402; the revised S9A and S9B Fig).

Minor points:

1. It seems to be difficult for readers to understand Fig.2A, so more detail descriptions or explanations are needed.

Thanks for this good point. Based on the Pot1$_{DBD}$-Tel18 structure, we generated the Pot1$_{DBD}$-ssDNA structural models with 0-3 nucleotide linkers. Although energy minimization was not applied in the modeling, the structural models were generated based on tight conformational and stereochemical constraints on both DNAs and proteins. In the revised manuscript, following this reviewer's suggestion, we have described this structural modelling in more details (Pages 9 lines 181-184), and moved the panel of the modeling data from original Fig 2 to revised supplementary Figure S4 (revised S4 Fig).

2. In page 16 lines 307 and 316, I am afraid that alpha 1 must be alpha 2, and alpha 2 must be alpha 3. Please confirm this point.

Corrected.

3. In page 26 lines 506-9, it is hard to understand why there is a description of GST-based purification here.

For preparation of the Pot1$_{OB3}$-Tpz1$_{PIM}$ and Tpz1$_{CBM}$-Ccq1$_{TAD}$ complexes, Pot1$_{OB3}$ and Ccq1$_{TAD}$ were respectively cloned into a modified pET28a vector with a SUMO protein fused at the N terminus after the 6×His tag. Tpz1$_{PIM}$ and Tpz1$_{CBM}$ were respectively cloned into a modified pGEX vector with a GST tag. Thus, for purification of the protein samples of the Pot1$_{OB3}$-Tpz1$_{PIM}$ and Tpz1$_{CBM}$-Ccq1$_{TAD}$ complexes, we performed a two-step affinity purification scheme (His and GST purification).

4. In page 29 lines 574, the reference #60 is not consistent with the description as "Moser et al, 2015" in S2 Table.

We apologize for this mistake, and we have corrected it in the revised S2 Table.

5. In page 29 lines 577, kanMx6 must be kanMX6.

Corrected.

6. In page 43 lines 949, EcoRI must be EcoRI (R should be non-italic).

Corrected.

7. the reference #60 is not consistent with the description as "Moser et al, 2015" in S2 Table.

Corrected it in the revised S2 Table.
